# Elevated expression of prostate cancer-associated genes is linked to down-regulation of microRNAs

**DOI:** 10.1186/1471-2407-14-82

**Published:** 2014-02-11

**Authors:** Kati Erdmann, Knut Kaulke, Cathleen Thomae, Doreen Huebner, Mildred Sergon, Michael Froehner, Manfred P Wirth, Susanne Fuessel

**Affiliations:** 1 Department of Urology, University Hospital Carl Gustav Carus, Fetscherstrasse 74, 01307 Dresden, Germany; 2 Institute of Pathology, University Hospital Carl Gustav Carus, Fetscherstrasse 74, 01307 Dresden, Germany

**Keywords:** Biomarkers, Alpha-methylacyl-CoA racemase (AMACR), Enhancer of zeste homolog 2 (EZH2), microRNAs, miR-186, miR-26a, Prostate cancer

## Abstract

**Background:**

Recent evidence suggests that the prostate cancer (PCa)-specific up-regulation of certain genes such as AMACR, EZH2, PSGR, PSMA and TRPM8 could be associated with an aberrant expression of non-coding microRNAs (miRNA).

**Methods:**

*In silico* analyses were used to search for miRNAs being putative regulators of PCa-associated genes. The expression of nine selected miRNAs (hsa-miR-101, -138, -186, -224, -26a, -26b, -374a, -410, -660) as well as of the aforementioned PCa-associated genes was analyzed by quantitative PCR using 50 malignant (Tu) and matched non-malignant (Tf) tissue samples from prostatectomy specimens as well as 30 samples from patients with benign prostatic hyperplasia (BPH). Then, correlations between paired miRNA and target gene expression levels were analyzed. Furthermore, the effect of exogenously administered miR-26a on selected target genes was determined by quantitative PCR and Western Blot in various PCa cell lines. A luciferase reporter assay was used for target validation.

**Results:**

The expression of all selected miRNAs was decreased in PCa tissue samples compared to either control group (Tu *vs* Tf: -1.35 to -5.61-fold; Tu *vs* BPH: -1.17 to -5.49-fold). The down-regulation of most miRNAs inversely correlated with an up-regulation of their putative target genes with Spearman correlation coefficients ranging from -0.107 to -0.551. MiR-186 showed a significantly diminished expression in patients with non-organ confined PCa and initial metastases. Furthermore, over-expression of miR-26a reduced the mRNA and protein expression of its potential target gene AMACR *in vitro*. Using the luciferase reporter assay AMACR was validated as new target for miR-26a.

**Conclusions:**

The findings of this study indicate that the expression of specific miRNAs is decreased in PCa and inversely correlates with the up-regulation of their putative target genes. Consequently, miRNAs could contribute to oncogenesis and progression of PCa via an altered miRNA-target gene-interaction.

## Background

Prostate cancer (PCa) is the second most frequent tumor and the sixth leading cause of cancer-related death among males worldwide [1]. Even though early detection of PCa has dramatically increased since the introduction of serum prostate-specific antigen (PSA) measurement, the lack of specificity of PSA as a tumor marker results in a high rate of unnecessary biopsies [2]. Consequently, various attempts have been made to identify new biomarkers that allow the detection of PCa at an early stage as well as the discrimination between benign and malignant alterations of the prostate. In previous studies, we have analyzed selected transcript markers such as AMACR, EZH2, PSGR, PSMA and TRPM8 among others in PCa tissue specimens. All of these markers were significantly up-regulated in PCa tissue compared to non-malignant prostate tissue and thus, could be of clinical importance for diagnostic purposes [3-6].

Originally identified as an enzyme that is involved in the metabolism of fatty acids AMACR (alpha-methylacyl-CoA racemase) is also highly over-expressed in PCa and its immunohistochemical detection is currently used by pathologists to achieve definitive diagnosis of PCa [7,8]. It has been shown that AMACR can modify the growth of PCa cells in an androgen-independent manner [9]. EZH2 (enhancer of zeste homolog 2) is a member of the polycomb-group family and functions as a transcriptional repressor [10]. As an oncogene it is frequently up-regulated in hormone-refractory metastatic PCa suggesting a critical role for EZH2 in disease progression [11]. PSGR (prostate-specific G-protein coupled receptor; synonym: olfactory receptor, family 51, subfamily E, member 2 (OR51E2)) is a member of the G-protein-coupled olfactory receptor family that is predominantly expressed in the human prostate [12,13]. PSGR has been described to be over-expressed in PCa tissue [13,14] and a multiplexed model based on the detection of PSGR and PCA3 (prostate cancer gene 3) in urine improved the specificity for PCa prediction [15]. PSMA (prostate-specific membrane antigen; synonym: folate hydrolase 1 (FOLH1)) is a cell-surface antigen with abundant and virtually universal expression in PCa which increases as the cancer progresses [16,17]. Since PSMA is an antigen that is highly specific for PCa tissue its targeting can be used for *in vivo* imaging and immunotherapy of PCa [18,19]. TRPM8 (transient receptor potential cation channel, subfamily M, member 8; synonym: Trp-p8) is involved in the regulation of the intracellular Ca^2+^ concentration and exhibits an elevated expression in PCa [20,21]. TRPM8 is an androgen-responsive gene and essential for the survival of PCa cells [22].

The tumor-specific up-regulation of the aforementioned genes suggests a functional role for these genes in the development and progression of PCa. However, the genetic and epigenetic mechanisms that lead to their up-regulation are mainly unknown. The demonstrated abnormal expression patterns could be associated with a deregulation of microRNA (miRNA) expression. MiRNAs are small (~22 nucleotides) non-coding RNAs that are involved in a variety of oncogenic pathways [23]. As post-transcriptional regulators they bind to the 3′-untranslated region (3′UTR) of their target mRNA resulting in either translational repression or mRNA degradation [23,24]. Depending on their target genes miRNAs can either function as oncogenes or tumor-suppressors [24].

It has been reported that miRNAs have distinct expression profiles in various human cancers [25-27]. Several profiling studies have also shown that the expression of miRNAs is commonly altered in PCa compared to normal tissues [25,28-33]. A deregulation of the miRNA expression consequently leads to an altered interaction with their respective mRNA targets and thus, promotes abnormal cellular functions [34,35]. To evaluate the influence of miRNAs on the onset or progression of PCa it is therefore of utmost importance to identify and analyze potential interactions between PCa-associated genes and their putative miRNA regulators. However, only few studies have assessed such a connection between a miRNA deregulation and an up-regulation of PCa-specific genes. Of the PCa-associated genes investigated in this study a miRNA-mediated regulation has been reported only for EZH2 so far [36-40].

The aim of this study was to identify miRNAs that could potentially regulate the expression of genes that are known to be up-regulated in PCa. Subsequently, the expression levels of both the candidate miRNAs and the PCa biomarkers were analyzed in malignant and non-malignant prostate tissues. Furthermore, the miRNA expression data were evaluated with regard to a potential correlation with the expression levels of the PCa-associated genes as well as with clinicopathological parameters. In an initial assessment the influence of exogenously administered miR-26a on the mRNA and protein expression of its known target EZH2 as well as its potential new target gene AMACR was investigated in various PCa cell lines. Subsequently, target validation for miR-26a was conducted by a luciferase reporter assay.

## Methods

### *In silico* miRNA prediction

To identify miRNAs that might target the PCa-associated genes AMACR, EZH2, PSGR, PSMA, and TRPM8 the following publicly available bioinformatic prediction programs as well as a database of experimentally supported miRNA targets were used: TargetScanHuman v5.1, TargetScanS, PicTar (based on conservation in mammals), MicroCosm Targets, microRNA.org (release 03/2009), Human miRNA Targets (optimized intersection: PicTar, TargetScanS), DIANA microT v3.0 and DIANA TarBase v5.0 (Additional file 1: Table S1). For subsequent analyses miRNAs were considered that were predicted (i) by multiple algorithms per gene or (ii) for more than one gene.

### Tissue specimens

Fresh-frozen malignant (tumor: Tu) and corresponding non-malignant (tumor-free: Tf) specimens from 50 patients with primary PCa who underwent radical prostatectomy as well as 30 samples from patients with benign prostatic hyperplasia (BPH) were used for mRNA and miRNA expression analyses. The BPH samples were obtained from patients undergoing radical cystectomy for bladder cancer or prostatic adenomectomy for BPH treatment. None of the PCa patients received neoadjuvant hormonal treatment. The clinicopathological data of the patients are given in Table 1. After removal of the prostate gland, the tissue was roughly cut into regions based on its normal and tumor suspicious appearance and then cryo-preserved in liquid nitrogen. For further analyses, cryosections of available tissues were prepared and the tumor cell amount of all samples was estimated by an experienced pathologist on hematoxylin-eosin stained serial tissue sections (start, middle, end). The tumor cell amount of the Tu samples was ≥50% and that of Tf and BPH samples 0%. Tissue collection and analysis was approved by the internal review board of the Technical University of Dresden (EK194092004 and EK195092004). Written informed consent was obtained from each patient.

**Table 1:** Clinicopathological data of the patients

**Parameter**	**PCa**	**BPH**
**Total patient number**	50	30
**Age at surgery [years]**		
Median (range)	65 (49 – 78)	72 (50 – 86)
**Pre-operative PSA [ng/ml]**		
Median (range)	10.2 (2.8 – 113.0)	2.6 (0.2 – 46.2)
	**n (%)**	
**Tumor stage**		
pT2 (organ-confined)	23 (46%)	-
pT3 + 4 (nonorgan-confined)	27 (54%)	-
**Gleason score**		
< 7 (low)	16 (32%)	-
7 (intermediate)	19 (38%)	-
> 7 (high)	15 (30%)	-
**Lymph node status**		
N0	43 (86%)	-
N+	7 (14%)	-
**Distant metastases at prostatectomy**		
M0	46 (92%)	-
M+	4 (8%)	-
**Initial metastases**		
N0M0	40 (80%)	-
N+/M+	10 (20%)	-

### Cell lines

The human PCa cell lines DU-145 (HTB-81), PC-3 (CRL-1435) and LNCap (CRL-1740) were obtained from the American Type Culture Collection (ATCC) and maintained at standard conditions (37°C, humidified atmosphere containing 5% CO_2_) without antibiotics. DU-145 and PC-3 cells were cultured in DMEM (4.5 g/l glucose) supplemented with 10% fetal bovine serum (FBS), 1% 1 M HEPES buffer and 1% MEM non-essential amino acids, whereas LNCap cells were grown in RPMI-1640 including 10% FBS and 1% MEM non-essential amino acids (all from Life Technologies).

### MiRNA mimics, siRNAs and transient transfection

The mimic for miR-26a (PM10249) and the Pre-miR Negative Control #1 (miR-CON) were obtained from Life Technologies. Specific small interfering RNAs (siRNAs) directed against AMACR (siR-AMACR; sense: GAGAUUUAUCAGCUUAACU, antisense: AGUUAAGCUGAUAAAUCUC) and EZH2 (siR-EZH2; sense: CACAAGUCAUCCCAUUAAA, antisense: UUUAAUGGGAUGACUUGUG) as well as the negative control siRNA (siR-CON; SR-CL000-005) were synthesized by Eurogentec. Cells were washed with PBS and transfected for 4 h in serum-free OptiMEM (Life Technologies) using DOTAP liposomal transfection reagent (Roche) according to the manufacturer’s instructions. The final concentrations of the transfectants and their respective controls were either 100 nM (miRNA mimic) or 150 nM (siRNAs). After 4 h, transfection medium was replaced by fresh cell culture medium and cells were incubated for another 48 h. For further analyses cells were then harvested by trypsin/EDTA treatment.

### RNA isolation and cDNA synthesis

RNA was isolated from cells using peqGOLD TriFast (Peqlab) and from tissue cryosections either using Invisorb Spin Tissue RNA Mini Kit (Invitek; for subsequent mRNA analysis) or peqGOLD TriFast (for subsequent miRNA analysis) according to the manufacturers’ recommendations. For mRNA analysis in tissues and cells, reverse transcription of 500 ng RNA into cDNA was carried out using SuperScript II Reverse Transcriptase (Life Technologies) and random hexamer primers (GE Healthcare) according to the manufacturers’ recommendations. For miRNA analysis in tissue samples, a total of 400 ng RNA was reverse transcribed into cDNA using the TaqMan MicroRNA Reverse Transcription Kit and Megaplex RT Primers (Human Pool A; both Life Technologies) which allows for reverse transcription of up to 381 miRNAs in a single reaction.

### Quantitative polymerase chain reaction (qPCR)

Gene expression of AMACR, EZH2, PSGR, PSMA and TRPM8 as well as of the reference gene TBP (TATA box binding protein) was measured by qPCR using the LightCycler FastStart DNA Master Hybridization Probes Kit and the LightCycler 1.5 instrument (both Roche). Primers and probes are listed in Additional file 1: Table S2; qPCR conditions are summarized in Additional file 1: Table S3. The mRNA copy number of a single marker was calculated in relation to the amplification product amounts of external standards as described previously [3]. All qPCR measurements were carried out at least twice as independent PCR runs for each cDNA sample. Samples were measured for a third time if differences of >30% occurred. The means of all measurements were used for further calculations. Relative expression levels of PCa related markers were obtained by normalization to the reference gene TBP.

The expression of the selected miRNAs was quantified by miRNA-specific TaqMan MicroRNA Assays (Life Technologies) according to the manufacturer’s instructions using the TaqMan Gene Expression Master Mix and the LightCycler 480 instrument (both Roche) (Additional file 1: Table S3). The following TaqMan MicroRNA Assays were used: 002253 (hsa-miR-101), 002284 (hsa-miR-138), 002285 (hsa-miR-186), 002099 (hsa-miR-224), 000405 (hsa-miR-26a), 000407 (hsa-miR-26b), 000563 (hsa-miR-374a), 001274 (hsa-miR-410), 001515 (hsa-miR-660) and 001006 for the reference RNA (RNU48). RNU48 was selected for normalization purposes due to its reported biological stability and its usefulness as a reference molecule for miRNA expression analyses in PCa and other cancer tissues [41-43]. Automatic second derivative analysis was applied for the determination of the crossing points (CP). Each CP was determined twice in independent qPCR runs and the mean value was used for further calculations. If the mean deviation of both CP values exceeded 0.25, a third measurement was done and included in the calculation of the mean. Standard curves were used to determine the copy number of a single miRNA. Relative expression levels of the miRNAs were obtained by normalization to the reference RNA RNU48.

In tissue samples the fold expressions of PCa-associated genes as well as of miRNAs were determined relative to the median relative expression in Tf or BPH tissues. For transfection experiments the fold expressions were calculated using the ΔΔCP method.

### Heat map generation

Heat map generation was carried out using the Genesis software package. Relative expression data were log-transformed and fully normalized for genes and miRNAs.

### Western Blot analysis

Protein separation and subsequent Western blotting were performed as described previously [44]. Membranes were probed with primary antibodies against AMACR (1:1000; Cell Signaling, clone 2A10), EZH2 (1:750; Cell Signaling, clone AC22) and α-tubulin (1:5000; Calbiochem, clone DM1A); the latter served as a loading control. The secondary polyclonal rabbit anti-mouse immunoglobulin HRP-linked antibody (1:1000; Dako, P0260) as well as the Enhanced Chemiluminescence Kit (GE Healthcare) were used for visualization. Quantification of the protein content was performed by means of computer-assisted videodensitometry (Quantity One Basic, Bio-Rad).

### Construction of plasmid vectors and luciferase reporter assay

A putative binding site of miR-26a within the 3′UTR of AMACR was identified using the target prediction tool of microRNA.org (Additional file 1: Table S1). To construct luciferase reporter vectors, oligonucleotides (Biomers) comprising the wildtype or mutated binding site were inserted downstream of the *Firefly* luciferase gene into the pmir-GLO Dual-Luciferase miRNA Target Expression Vector (Promega) according to the manufacturer’s instructions. The insert regions in the vectors were sequenced (GATC Biotech) to verify incorporation of the respective target sequence. The resulting vectors are referred to as pmir-GLO-A26a (AMACR-specific miRNA-26a-binding sequence; AAC ACA CTG AGG AGA TAC TTG AA) and pmir-GLO-Amut26a (mutated AMACR-specific miRNA-26a-binding sequence; AAC ACA CTG AGG **C**GA **G**AC **CCA** AA). Nucleotides in bold indicate changes introduced within the target sequence to generate the mutant form.

For luciferase reporter assays, DU-145 cells were cultured in 24-well plates and co-transfected with 1.5 μg of the indicated vector and 100 nM of miR-26a mimic or miR-CON using Lipofectamine 2000 (final concentration 20 ng/μl; Life Technologies) for 24 h. Following incubation with fresh cell culture medium for another 24 h, cells were lysed and analyzed for luciferase activity using the Dual-Glo Luciferase Assay System (Promega) and a Mithras LB 940 Multimode Microplate Reader (Berthold) according to the manufacturers’ instructions. Following background adjustment, *Firefly* luciferase activity was normalized to *Renilla* luciferase activity. The normalized luciferase activity was then compared to that of the pmir-GLO-A26a vector co-transfected with miR-CON. For each transfection, luciferase activity was averaged from three replicates.

### Statistics

Statistical analyses were carried out with the PASW Statistics 18.0.0 (SPSS) software. Correlations were assessed by Spearman’s rank correlation coefficients. Group comparisons were conducted as indicated. A p value <0.05 was defined to be statistically significant; p < 0.1 was considered as a statistical trend.

## Results

### Up-regulation of PCa-associated genes

The expression levels of the PCa-associated genes AMACR, EZH2, PSGR, PSMA, and TRPM8 were analyzed in 50 Tu and corresponding Tf prostate tissue specimens as well as in 30 BPH tissue samples. The median expression levels of all genes were significantly higher in Tu tissue compared to either control group with median fold expressions ranging from +1.61 to +19.36 *versus* Tf tissue and from +3.02 to +36.65 *versus* BPH tissue (Table 2). The tissue type-dependent expression of the genes was further highlighted in a heat map (Additional file 1: Figure S1), whereupon the clearest expression differences could be seen between Tu and BPH tissues. The highest relative transcript level was observed for AMACR and the lowest for EZH2 regardless of the tissue specimen subset. Compared to either control tissue the highest up-regulation in Tu tissue was detected for AMACR (+19.36 *vs* Tf; +36.65 *vs* BPH), whereas the lowest was observed for EZH2 (+1.61 *vs* Tf; +3.02 *vs* BPH). Furthermore, the percentages of Tu samples with an unaltered, up-regulated or down-regulated fold expression compared to either control group was evaluated for each gene (Additional file 1: Table S4). For this purpose a fold expression of ≥2.0 was considered as up-regulation and of ≤ −2.0 as down-regulation, whereas the remaining fraction was regarded as an unaltered expression. Compared to Tf samples, more than 40% of the Tu samples showed an up-regulation for each gene with the highest rate of up-regulation (80%) for AMACR. A down-regulated or unaltered expression was observed for 2-18% and 10-58% of the Tu samples, respectively. Compared to the BPH group, 80-90% of the Tu samples showed an up-regulation of the respective genes, whereas only minor proportions (≤20%) exhibited an unaltered or diminished expression.

**Table 2:** Differentially expressed genes between malignant and non-malignant prostate tissues samples

**Gene**	**Median relative transcript levels**			**Median fold expressions**	
	**Tu**	**Tf**	**BPH**	**Tu *vs *** **Tf**_**[median]**_	**Tu *vs *** **BPH**_**[median]**_
	**(n = 50)**	**(n = 50)**	**(n = 30)**		
AMACR	2093.38	108.14	57.12	+19.36**	+36.65**
EZH2	0.93	0.58	0.31	+1.61**	+3.02**
PSGR	44.70	16.72	2.45	+2.67**	+18.23**
PSMA	28.02	11.47	1.88	+2.44**	+14.91**
TRPM8	36.58	13.44	4.01	+2.72**	+9.12**

Depicted are the median relative transcript levels of the PCa-associated genes (normalized to TBP) in Tu, matched Tf and BPH tissue samples as well as the median fold expressions of the Tu samples compared to the indicated control group. P values were calculated by the Mann–Whitney *U* test with a two-sided 95% confidence interval (**p < 0.01).

### Identification of putative miRNA regulators for PCa-associated genes

*In silico* miRNA prediction identified numerous putative miRNA regulators for each PCa-associated gene. The resulting miRNA pool was then filtered for possibly relevant miRNAs according to the aforementioned selection criteria. In this manner, nine candidate miRNAs were selected from the entire miRNA pool for subsequent qPCR analyses (Table 3): hsa-miR-101, -138, -186, -224, -26a, -26b, -374a, -410 and -660. The most frequent predictions were observed for miR-101, miR-138, miR-26a and miR-26b for EZH2.

**Table 3:** **Results of the *in silico *** **analyses and the Spearman rank correlation**

**miRNA**	**Target gene**				
	**AMACR**	**EZH2**	**PSGR**	**PSMA**	**TRPM8**
miR-101		8x			
		−0.156^#^			
miR-138	1x	7x			
	−0.256**	−0.215*			
miR-186	2x			4x	
	−0.551**			−0.418**	
miR-224	4x				
	−0.384**				
miR-26a	1x	6x		1x	3x
	−0.335**	−0.383**		−0.237**	−0.362**
miR-26b	1x	6x			3x
	−0.154^#^	−0.141			−0.107
miR-374a			2x	3x	3x
			−0.230**	−0.248**	−0.329**
miR-410	1x	1x	2x	2x	1x
	−0.273**	−0.293**	−0.283**	−0.239**	−0.395**
miR-660	3x			3x	
	−0.241**			−0.289**	

In the upper rows the respective numbers of predictions out of eight possible algorithms are denoted. In the lower rows results of the Spearman rank correlation expressed as Spearman correlation coefficients are displayed. The relative transcript levels of the target genes and the miRNAs in all tissue samples (Tu, Tf, BPH) were used for the calculation of the Spearman correlation coefficients. Empty cells indicate that there was no prediction by any algorithm in the *in silico* analyses. *p < 0.05, **p < 0.01, ^#^p < 0.1 (statistical trend).

### Down-regulation of selected miRNAs in PCa tissues

The expression levels of these nine miRNAs were then determined in the same sample cohort used for the gene expression analyses. Due to loss of tissue during processing only 46 Tf tissue specimens were available for the miRNA expression analyses. The median expression levels of all miRNAs were lower in Tu tissue compared to Tf and BPH tissue (Table 4). This was further emphasized in a heat map based on the relative miRNA expression in the various prostate tissue specimens (Additional file 1: Figure S1), whereupon the most distinct expression differences occurred between Tu and BPH tissue samples. The lowest relative transcript level was observed for miR-410 and the highest for miR-26a in all tissue subsets. For all miRNAs the tumor-specific down-regulation was statistically significant compared to Tf tissue samples with median fold expressions ranging from -1.35 to -5.61. Except for miR-101 and miR-26b the observed decrease in expression was also significant when compared to BPH tissue samples with median fold expressions ranging from -1.17 to -5.49. Compared to either control tissue the highest down-regulation in Tu tissue was detected for miR-138 (-5.61 *vs* Tf; -5.49 *vs* BPH), while the lowest ratio of down-regulation was observed for miR-26b (-1.35 *vs* Tf; -1.17 *vs* BPH). The proportions of Tu samples with an unaltered, up-regulated or down-regulated fold expression compared to either control group are summarized in Additional file 1: Table S4. Compared to Tf samples, a high percentage of down-regulation (54-86%) was observed for most of the miRNAs except for miR-101 (44%), miR-26b (32%) and miR-660 (38%). Only minor fractions of the Tu samples (≤10%) showed an up-regulation. Depending on the miRNA the fraction of Tu samples with an unaltered expression was 14-60%. Compared to the BPH samples, the lowest proportions of down-regulated Tu samples were observed for miR-101 (26%) and miR-26b (24%). For the other miRNAs the distributions of fold expression did not shift at all or shifted only marginally from the distribution pattern compared to the Tf group. The highest down-regulation rate compared to either control group was observed for miR-186 with none of the Tu samples showing an up-regulation.

**Table 4:** Differentially expressed miRNAs between malignant and non-malignant prostate tissues samples

**miRNA**	**Median relative transcript levels (x10**^**-3**^**)**			**Median fold expressions**	
	**Tu**	**Tf**	**BPH**	**Tu *vs *** **Tf**_**[median]**_	**Tu *vs *** **BPH**_**[median]**_
	**(n = 50)**	**(n = 46)**	**(n = 30)**		
miR-101	1.52	2.91	2.01	−1.92**	−1.32
miR-138	0.19	1.08	1.06	−5.61**	−5.49**
miR-186	39.52	138.59	160.43	−3.51**	−4.06**
miR-224	1.19	4.12	3.22	−3.45**	−2.70**
miR-26a	179.04	380.81	310.84	−2.13**	−1.74**
miR-26b	51.77	69.98	60.60	−1.35 *	−1.17
miR-374a	17.63	39.65	33.84	−2.25**	−1.92**
miR-410	0.08	0.28	0.33	−3.64**	−4.29**
miR-660	13.46	22.50	22.63	−1.67**	−1.68**

Depicted are the median relative transcript levels of the evaluated miRNAs (normalized to RNU48) in Tu, matched Tf and BPH tissue samples as well as the median fold expressions of the Tu samples compared to the indicated control group. P values were calculated by the Mann–Whitney *U* test with a two-sided 95% confidence interval (*p < 0.05, **p < 0.01).

### Expression levels of PCa-associated genes and selected miRNAs depending on clinicopathological parameters

Furthermore, the relative transcript levels of the relevant genes as well as of the selected miRNAs were compared with regard to the different clinicopathological parameters. With the exception of TRPM8 none of the relevant genes showed a significant association with age, serum PSA concentration, tumor stage, Gleason score or initial metastases at prostatectomy (Additional file 1: Table S5). Solely the expression of TRPM8 was with 25.14 significantly lower in patients with nonorgan-confined tumors (pT3 + 4, n = 27) compared to organ-confined tumors (pT2, n = 23) with 52.44 (p = 0.03) (Additional file 1: Table S5).

**Figure 1:**
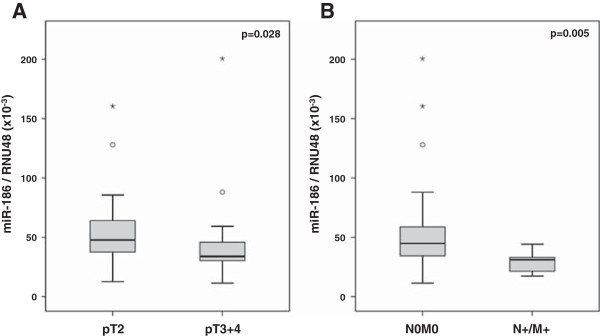
**Relative transcript levels of miR-186 in PCa samples with regard to clinicopathological parameters. (A)** Comparison of the miR-186 expression levels in organ-confined (pT2, n = 23) *versus* nonorgan-confined tumors (pT3 + 4, n = 27). **(B)** Comparison of the miR-186 expression levels in patient samples without any initial metastases (N0M0, n = 40) *versus* those with initial metastases (N+/M+, n = 10). Transcript levels of miR-186 were normalized to RNU48. P values were calculated by the Mann–Whitney *U* test with a two-sided 95% confidence interval. Outliers are indicated by circles; extreme values are indicated by asterisks.

Regarding the expression of the selected miRNAs in relation to the clinicopathological parameters significant associations were only observed for miR-186. The relative expression of miR-186 was with 33.89×10^-3^ significantly lower in nonorgan-confined tumors (pT3 + 4, n = 27) compared to organ-confined tumors (pT2, n = 23) with 47.70×10^-3^ (p = 0.028; Figure 1A & Additional file 1: Table S6). Patients with initial metastases (N+/M+, n = 10) displayed also a significantly lower transcript level of miR-186 than patients without any initial metastases (N0M0, n = 40): 44.86×10^-3^ for N0M0 *vs* 31.18×10^-3^ for N+/M+, p = 0.005 (Figure 1B & Additional file 1: Table S6). No significant associations were observed between the expression of any miRNA and age, serum PSA concentration or Gleason score (Additional file 1: Table S6). However, the transcript levels of miR-138 and miR-224 were also frequently lower albeit not significantly in tumors that were more aggressive or in an advanced disease stage (Additional file 1: Table S6). Furthermore, the expression of miR-26a, miR-374a and miR-410 gradually decreased with increasing Gleason score (Additional file 1: Table S6).

### Correlation between the expression of PCa-associated genes and their putative miRNA regulators

A possible correlation between the expression of the selected miRNAs and of their putative target genes was analyzed by Spearman rank correlation using the expression data gained from all tissue specimens (50 Tu, 46 Tf, 30 BPH). The expression levels of specific miRNAs showed weak to moderate inverse correlations with the expression levels of their putative target genes. The Spearman correlation coefficients (r_s_) ranged from -0.107 to -0.551 (Table 3). Except for miR-101 and miR-26b these correlations were statistically significant. However, a statistical trend was found for the combinations miR-101/EZH2 (r_s_ = -0.156, p = 0.081) and miR-26b/AMACR (r_s_ = -0.154, p = 0.086). Overall, the strongest correlations with the expression of their putative target genes were observed for miR-186, miR-26a and miR-224 (Table 3).

**Figure 2:**
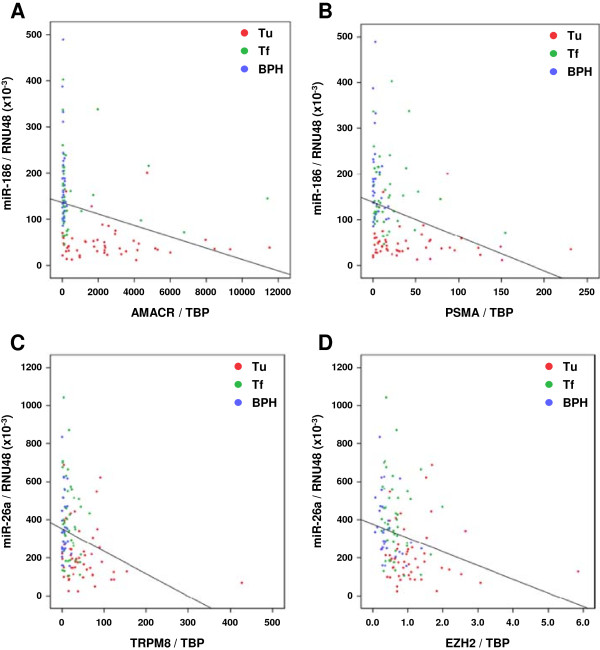
**Matched miRNA and target gene expression with regard to the prostate tissue specimens.** Depicted are exemplary scatter plots with trend lines for **(A)** miR-186/AMACR, **(B)** miR-186/PSMA, **(C)** miR-26a/TRPM8 and **(D)** miR-26a/EZH2. Transcript levels of miRNAs and target genes were normalized to RNU48 and TBP, respectively.

Exemplary scatter plots based on the matched miRNA and target gene expression in all three tissue subsets are shown in Figure 2 for the combinations miR-186/AMACR (r_s_ = -0.551, p < 0.01), miR-186/PSMA (r_s_ = -0.418, p < 0.01), miR-26a/TRPM8 (r_s_ = -0.362, p < 0.01) and miR-26a/EZH2 (r_s_ = -0.383, p < 0.01). These scatter plots demonstrate that tumor tissues (Tu: red dots) exhibited a higher expression of the relevant genes which in turn was associated with a rather low miRNA expression. In contrast, a high miRNA expression in the non-malignant control groups (Tf: green dots; BPH: blue dots) was linked to a lower expression of the relevant genes. This pattern of differential miRNA/gene expression was also observed for all the other predicted miRNA/gene combinations in the scatter plots (data not shown).

### Effects of exogenous miR-26a on the expression of selected target genes in PCa cell lines

Among the miRNAs studied here, miR-26a has already been identified as a direct regulator of EZH2 [36,38]. In the present study, miR-26a was also recognized as a putative regulator of AMACR. AMACR and to a smaller extent EZH2 are strongly expressed in the PCa cell lines DU-145, PC-3 and LNCap (data not shown). Furthermore, miR-26a was detectable in all three cell lines with DU-145 cells exhibiting the lowest expression of this miRNA (Table 5).

**Table 5:** Transcript expression of miR-26a in PCa cell lines

**Treatment**	**Median relative transcript levels (x10**^**-3**^**)**		
	**DU-145**	**PC-3**	**LNCap**
Untreated	35.1 ± 12.3	44.0 ± 29.8	66.7 ± 37.1
miR-CON (100 nM)	36.6 ± 14.4	33.2 ± 23.1	52.2 ± 36.3
miR-26a (100 nM)	30895.2 ± 13836.0^α,β^	16047.6 ± 13441.3^α,β^	11042.1 ± 6940.7^α,β^

The data represent the mean relative transcript levels of miR-26a (normalized to RNU48) of five independent experiments with their mean deviation in untreated cells or following treatment with 100 nM miR-26a mimic or miR-CON. P values were calculated by the Mann–Whitney *U* test with Bonferroni correction (^α^p < 0.05 *vs* untreated, ^β^p < 0.05 *vs* miR-CON).

**Figure 3:**
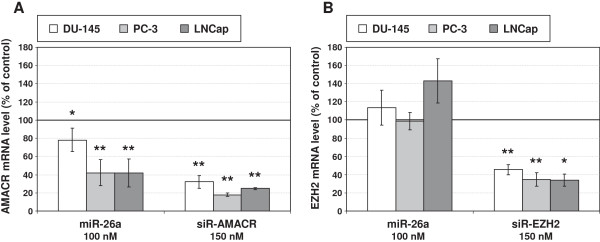
**Effect of miR-26a mimic and siRNAs on target gene mRNA expression in PCa cell lines.** Transcript levels of **(A)** AMACR and **(B)** EZH2 were determined by qPCR and normalized to TBP. Normalized values are shown relative to the corresponding control treatments (100%): miR-CON (100 nM) for treatment with miR-26a mimic and siR-CON (150 nM) for treatment with siRNAs, respectively. Values represent averages of two to five independent experiments with their mean deviation. A one-tailed paired t-test was used to compare differences between cells treated with miR-26a mimic or siRNAs and the respective control treated cells (miR-CON or siR-CON): *p < 0.05, **p < 0.01.

**Figure 4:**
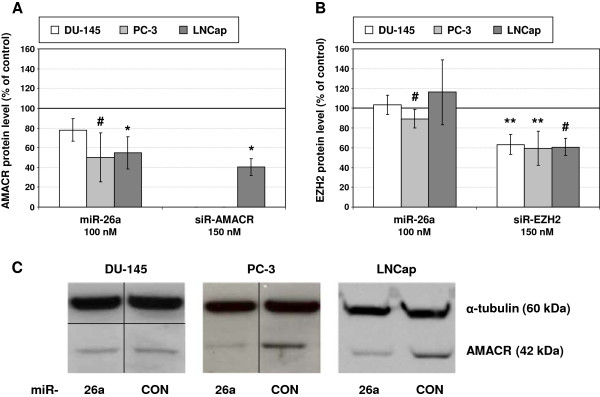
**Effect of miR-26a mimic and siRNAs on target gene protein expression in PCa cell lines.** Protein levels of **(A)** AMACR and **(B)** EZH2 were determined by Western Blot and normalized to α-tubulin. Normalized values are shown relative to the corresponding control treatments (100%): miR-CON (100 nM) for treatment with miR-26a mimic and siR-CON (150 nM) for treatment with siRNAs, respectively. Values represent averages of two to five independent experiments with their mean deviation. A one-tailed paired t-test was used to compare differences between cells treated with miR-26a mimic or siRNAs and the respective control treated cells (miR-CON or siR-CON): *p < 0.05, **p < 0.01, ^#^p < 0.1 (statistical trend). **(C)** Exemplary Western Blots for the detection of AMACR protein following treatment with 100 nM miR-26a mimic or miR-CON are depicted. Alpha-tubulin served as loading control. Lines indicate that the sample lanes were not adjacent in the original gel. However, samples per cell line were retrieved from the same experiment and each sample was simultaneously probed for AMACR and α-tubulin.

In order to determine if miR-26a can influence the expression of its potential target genes AMACR and EZH2 PCa cells were transiently transfected with a miR-26a mimic. Specific siRNAs targeting AMACR or EZH2 were used as positive controls for the inhibition of the gene expression. Incubation of all three cells lines with the specific siRNAs led to notable reductions of the respective mRNA and protein levels (Figures 3 and 4). The siRNA against AMACR even produced a complete down-regulation of the AMACR protein in DU-145 and PC-3 cells.

Following exogenous administration of the miR-26a mimic a significant increase of this miRNA was observed in all three cell lines (Table 5). An over-expression of miR-26a diminished the AMACR transcript and protein level by about 20-60% and 20-50%, respectively, depending on the cell line (Figure 3A, Figure 4A, C). In contrast, treatment with the mimic for miR-26a did not produce a distinct inhibition of EZH2 mRNA and protein expression in any cell line (Figure 3B and 4B).

### Direct regulation of AMACR by miR-26a

**Figure 5:**
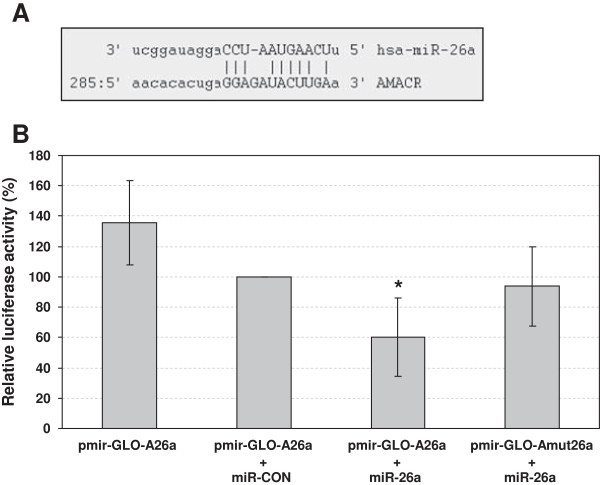
**miR-26a directly targets AMACR. (A)** The mature miR-26a sequence aligned with the predicted binding site within the 3′UTR of AMACR is depicted. **(B)** Luciferase activity was measured in DU-145 cells co-transfected with 100 nM miR-26a mimic and 1.5 μg of the wildtype (pmir-GLO-A26a) or mutated (pmir-GLO-Amut26a) luciferase reporter vector for AMACR. Normalized values are shown relative to the control treatment with 100 nM miR-CON and the wildtype luciferase reporter vector (100%). Values represent averages of six independent experiments with their mean deviation. A one-tailed paired t-test was used to compare differences between the respective treatments and the control treated cells (pmir-GLO-A26a + miR-CON): *p < 0.05.

To determine whether miR-26a can directly target the 3′UTR of AMACR, we studied the effects of the miR-26a mimic on a luciferase reporter vector containing the putative binding site for miR-26a within the 3′UTR of AMACR. The alignment of miR-26a with its putative target sequence within the human AMACR 3′UTR is depicted in Figure 5A. Since DU-145 cells exhibited the lowest expression of miR-26a, this cell line was used for the luciferase reporter assay. Co-transfection of DU-145 cells with the miR-26a mimic and the luciferase reporter vector containing the wildtype AMACR binding site led to a significant decrease in luciferase activity by about 40% compared to the control treatment (Figure 5B). There was no significant reduction in luciferase activity following co-transfection with miR-26a mimic and the luciferase reporter vector containing the mutated AMACR binding site (Figure 5B), thereby confirming that miR-26a can directly target AMACR.

## Discussion

It is widely accepted that oncogenesis and tumor progression is initiated through a deregulated expression of certain genes which then leads to the malignant transformation of the affected cells. In previous studies we have shown that genes such as AMACR, EZH2, PSGR, PSMA and TRPM8 are tumor-specifically up-regulated in PCa compared to benign tissue and thus, could be used for diagnostic purposes [3-6], whereupon the observed PCa-specific up-regulation of the relevant genes was comparable to those in the present study. Consequently, the elevated expression of the aforementioned genes can deeply impact the growth and survival of PCa cells eventually resulting in oncogenesis and/or tumor progression [9,11,13,22,45]. Therefore, such PCa-associated genes could represent suitable diagnostic tools as well as promising targets for the therapeutic intervention. However, the identification and characterization of the underlying mechanisms for the deregulation of these molecular markers are crucial for the understanding of the biology and clinical course of the disease. The demonstrated abnormal expression patterns could be associated with a deregulation of miRNAs which serve as post-transcriptional regulators of their target gene expression [23,24]. In accordance, miRNAs are aberrantly expressed in several types of cancers [25-27] and thus, could influence oncogenesis and tumor progression via an altered miRNA-target gene-interaction. Using *in silico* analyses nine miRNAs (miR-101, -138, -186, -224, -26a, -26b, -374a, -410, -660), which could potentially regulate the expression of the PCa-associated genes, have been selected for further analysis.

By using a qPCR approach it was revealed that the selected miRNAs are down-regulated in PCa compared to matched non-malignant tissue or BPH, respectively. Several studies have also demonstrated distinct miRNA expression profiles for PCa compared to normal prostate tissue [25,28-33]. However, a comparison of the studies by Ambs *et al.*[28], Volinia *et al.*[25] and Porkka *et al.*[30] showed that there are no overlapping subsets between the differentially expressed miRNAs analyzed in these studies [46]. The observed inconsistencies can mainly be attributed to different methods of tissue collection, RNA isolation and miRNA detection [47]. Therefore, a consensus on PCa-specific miRNA alterations has not been established to date.

The above mentioned discrepancies have also been observed for some of the miRNAs evaluated in this study. To begin with, some studies demonstrated a down-regulation for miR-101 [31], miR-224 [29], miR-26a [30], miR-26b [30] and miR-410 [28] in primary PCa samples compared to normal prostate tissue which is consistent with our results. In contrast to our data and to some of the aforementioned profiling studies, up-regulated expression levels in PCa tissues have been demonstrated for miR-101 [32], miR-26a [25,28] and miR-26b [31]. However, the results of the cited profiling studies were obtained by microarray or deep sequencing analysis and have not been validated by qPCR with the only exception of miR-26a which was confirmed to be up-regulated in a small subset of 10 prostatic tumors [28].

In agreement with our results and also based on an assessment by qPCR, a significant down-regulation in primary PCa compared to benign samples was noted for miR-101 [40], miR-26a [38] and miR-224 [43], whereas miR-26b was only diminished by trend [38]. In a small sample cohort, miR-138 was up-regulated in high grade tumors (Gleason score ≥8; n = 14) *versus* normal epithelium (n = 10), which is contradictory to our results [33]. Upon reviewing the current literature miR-186, miR-374a and miR-660 have not been demonstrated to be differentially expressed in primary PCa compared to benign prostate tissue to date. Therefore, this is the first study reporting that miR-186, miR-374a and miR-660 are significantly down-regulated in primary PCa compared with benign samples.

Furthermore, none of the profiling studies evaluated associations of the particular miRNAs with clinicopathological parameters or has further analyzed them with regard to the regulation of potential target genes [25,28-32]. Only in the qPCR-based study by Mavridis *et al.*, miR-224 expression was reported to be gradually decreased as Gleason score and tumor stage progressed and also to be associated with a favorable prognosis [43]. In the present study, the miR-224 transcript levels were also frequently lower albeit not significantly in tumors that were more aggressive or in an advanced disease stage. Eventually, a significant association with clinicopathological features was only observed for miR-186. A decreased miR-186 expression was significantly linked to more aggressive and advanced tumors indicating that down-regulation of miR-186 in PCa could be a factor of disease progression.

The present study also demonstrated that the deregulation of the miRNAs is linked to an increase of the transcript levels of their putative target genes. Except for miR-101 and miR-26b the expression levels of the evaluated miRNAs showed significantly weak to moderate inverse correlations with the expression levels of their putative target genes. Among the miRNAs included in this study miR-101 [36,40], miR-138 [37,39], miR-26a [36,38] and miR-26b [38] are of particular interest as some studies have already identified EZH2 as one of their direct target genes. This was also reflected here by the high prediction rate of these miRNAs for EZH2 in the *in silico* analyses. The link between these miRNAs and EZH2 has been demonstrated in numerous experimental settings amongst others investigating the importance of this regulatory mechanism for the onset and progression of various types of cancer including PCa. In various PCa cell lines, over-expression of miR-101, miR-26a and miR-26b could lead to repression of both EZH2 mRNA and protein as well as to a reduced cellular proliferation suggesting a tumor-suppressive function for these miRNAs in PCa [36,38,40].

For some initial continuative analysis, we focused on miR-26a as this miRNA has already been identified as a direct regulator of EZH2 in PCa [36,38]. Moreover, the down-regulated expression of miR-26a in clinical PCa samples has been shown to be significantly inversely correlated with EZH2 levels with a Spearman correlation coefficient of -0.516 (p = 0.0013) [38]. In the present study, there was also a significant inverse correlation between the expression of EZH2 and miR-26a (r_s_ = -0.383, p < 0.01). The differences between the two studies might be partly explained by the use of different sample cohorts. Koh *et al.* analyzed the expression of miR-26a and EZH2 in 36 prostate samples (18 Tu, 18 Tf) [38], whereas we conducted the expression analyses in a larger cohort of 126 prostate tissue samples (50 Tu, 46 Tf, 30 BPH) and thus, may have gained a higher statistical reliability. However, in the present study, miR-26a failed to decrease EZH2 when administered exogenously to PCa cells. This finding is similar to the results reported by the study of Cao *et al.* in which miR-26a reduced EZH2 protein levels only in DU-145 cells [36]. In contrast, Koh *et al.* reported that over-expression of miR-26a repressed both EZH2 mRNA and protein in DU-145, PC-3 and LNCap cells [38]. The authors attributed this discrepancy to methodical differences which cannot be excluded.

In addition to EZH2, AMACR was also identified as a target gene possibly regulated by miR-26a. The putative link between AMACR and miR-26a was reflected by a moderate inverse correlation of their expression levels in prostate tissue (r_s_ = -0.335, p < 0.01). *In vitro* results gathered in this study demonstrated that miR-26a can potently repress the mRNA and protein expression of AMACR depending on the cell line. A direct regulatory effect of miR-26a on the newly identified target gene AMACR was confirmed by luciferase reporter assay. Co-transfection of DU-145 cells with the miR-26a mimic and the luciferase reporter vector containing the wildtype AMACR binding site produced a decrease in luciferase activity by about 40%, whereas co-transfection with the luciferase reporter vector containing the mutated AMACR binding site did not lead to a reduction in luciferase activity. Taken together, this is the first study showing that the expression of AMACR can directly be regulated by a miRNA.

Overall, this is the first study that demonstrated potential interactions between the PCa-associated genes AMACR, PSGR, PSMA and TRPM8 and specific miRNAs. Notably, strong correlations were also observed between miR-186 and its putative target genes AMACR and PSMA as well as between miR-224 and its proposed target gene AMACR. Further research is warranted to confirm a direct regulatory effect of these miRNAs on their potential target genes.

## Conclusions

In conclusion, this study demonstrated that the expression of specific miRNAs is decreased in PCa and inversely correlates with the up-regulation of their putative target genes. Consequently, miRNAs could contribute to oncogenesis and progression of PCa via an altered miRNA-target gene-interaction. A preliminary *in vitro* assessment showed that exogenous administration of miR-26a resulted in a decreased expression of AMACR mRNA and protein depending on the cell line. By using a luciferase reporter assay, AMACR was confirmed as a direct target of miR-26a.

## Abbreviations

3′UTR: 3′-untranslated region; AMACR: Alpha-methylacyl-CoA racemase; BPH: Benign prostatic hyperplasia; CP: Crossing point; EZH2: Enhancer of zeste homolog 2; FBS: Fetal bovine serum; miRNA: microRNA; PCa: Prostate cancer; PCA3: Prostate cancer gene 3; PSA: Prostate-specific antigen; PSGR: Prostate-specific G-protein coupled receptor; PSMA: Prostate-specific membrane antigen; qPCR: Quantitative polymerase chain reaction; siRNA: small interfering RNA; TBP: TATA box binding protein; Tf: Tumor-free; TRPM8: Transient receptor potential cation channel, subfamily M, member 8; Tu: Tumor.

## Competing interests

The authors declare that they have no competing interests.

## Authors’ contributions

KE, KK, CT, DH and SF designed the experiments. KE, MPW and SF coordinated the study. MS and MF provided clinicopathological information. MPW contributed reagents, materials and analysis tools. KE, KK and CT performed the experiments and analyzed the data. KE, DH and SF drafted the manuscript. All authors read and approved the final manuscript.

## Pre-publication history

The pre-publication history for this paper can be accessed here:

http://www.biomedcentral.com/1471-2407/14/82/prepub

## Supplementary Material

Additional file 1: Figure S1 Heat map based on the relative expression of genes and miRNAs in human prostate tissue. Columns represent genes and miRNAs; rows represent prostate tissue samples (red squares: Tu samples, green squares: Tf samples, yellow squares: BPH samples). Transcript levels of genes and miRNAs were normalized to TBP and RNU48, respectively. **Table S1.** Web addresses of the bioinformatics resources used for miRNA prediction. **Table S2.** Sequences of primers and probes used for qPCR. **Table S3.** Conditions for qPCR measurements. **Table S4.** Distributions of fold expressions of PCa genes and miRNAs in prostate cancer. Depicted are the proportions of fold expression in Tu samples compared to either Tf or BPH samples. A fold expression of ≥2.0 was considered as up-regulation and of ≤ −2.0 as down-regulation, whereas the remaining proportion was regarded as an unaltered expression. **Table S5.** Expression of selected genes in prostate cancer dependent on clinicopathological parameters. Depicted are median relative transcript levels of the evaluated genes (normalized to TBP) in Tu tissue samples. A Mann–Whitney *U* test with a two-sided 95% confidence interval was used for two group comparisons. Three-group comparisons were carried out by the Kruskal–Wallis test followed by a Mann–Whitney *U* test with a two-sided 95% confidence interval for *post hoc* analyses. Significant differences are highlighted in bold. **Table S6.** Expression of selected miRNAs in prostate cancer dependent on clinicopathological parameters. Depicted are median relative transcript levels of the evaluated miRNAs (normalized to RNU48) in Tu tissue samples. A Mann–Whitney *U* test with a two-sided 95% confidence interval was used for two group comparisons. Three-group comparisons were carried out by the Kruskal–Wallis test followed by a Mann–Whitney *U* test with a two-sided 95% confidence interval for *post hoc* analyses. Significant differences are highlighted in bold. Click here for file
