# Supplementary material for: Elevated expression of prostate cancer-associated genes is linked to down-regulation of microRNAs
Source: BMC Cancer. 2014 Feb 11;14:82. doi: 10.1186/1471-2407-14-82 (PMC3923006; doi:10.1186/1471-2407-14-82)
Supplement: Additional file 1: Figure S1 — Heat map based on the relative expression of genes and miRNAs in human prostate tissue. Columns represent genes and miRNAs; rows represent prostate tissue samples (red squares: Tu samples, green squares: Tf samples, yellow squares: BPH samples). Transcript levels of genes and miRNAs were normalized to TBP and RNU48, respectively. Table S1. Web addresses of the bioinformatics resources used for miRNA prediction. Table S2. Sequences of primers and probes used for qPCR. Table S3. Conditions for qPCR measurements. Table S4. Distributions of fold expressions of PCa genes and miRNAs in prostate cancer. Depicted are the proportions of fold expression in Tu samples compared to either Tf or BPH samples. A fold expression of ≥2.0 was considered as up-regulation and of ≤ −2.0 as down-regulation, whereas the remaining proportion was regarded as an unaltered expression. Table S5. Expression of selected genes in prostate cancer dependent on clinicopathological parameters. Depicted are median relative transcript levels of the evaluated genes (normalized to TBP) in Tu tissue samples. A Mann–Whitney U test with a two-sided 95% confidence interval was used for two group comparisons. Three-group comparisons were carried out by the Kruskal–Wallis test followed by a Mann–Whitney U test with a two-sided 95% confidence interval for post hoc analyses. Significant differences are highlighted in bold. Table S6. Expression of selected miRNAs in prostate cancer dependent on clinicopathological parameters. Depicted are median relative transcript levels of the evaluated miRNAs (normalized to RNU48) in Tu tissue samples. A Mann–Whitney U test with a two-sided 95% confidence interval was used for two group comparisons. Three-group comparisons were carried out by the Kruskal–Wallis test followed by a Mann–Whitney U test with a two-sided 95% confidence interval for post hoc analyses. Significant differences are highlighted in bold. [file 1471-2407-14-82-S1.pdf]

Figure S1: Heat map based on the relative expression of genes and miRNAs in human prostate tissue. Columns represent genes and miRNAs; rows represent prostate tissue samples (red squares: Tu samples, green squares: Tf samples, yellow squares: BPH samples). Transcript levels of genes and miRNAs were normalized to TBP and RNU48, respectively.

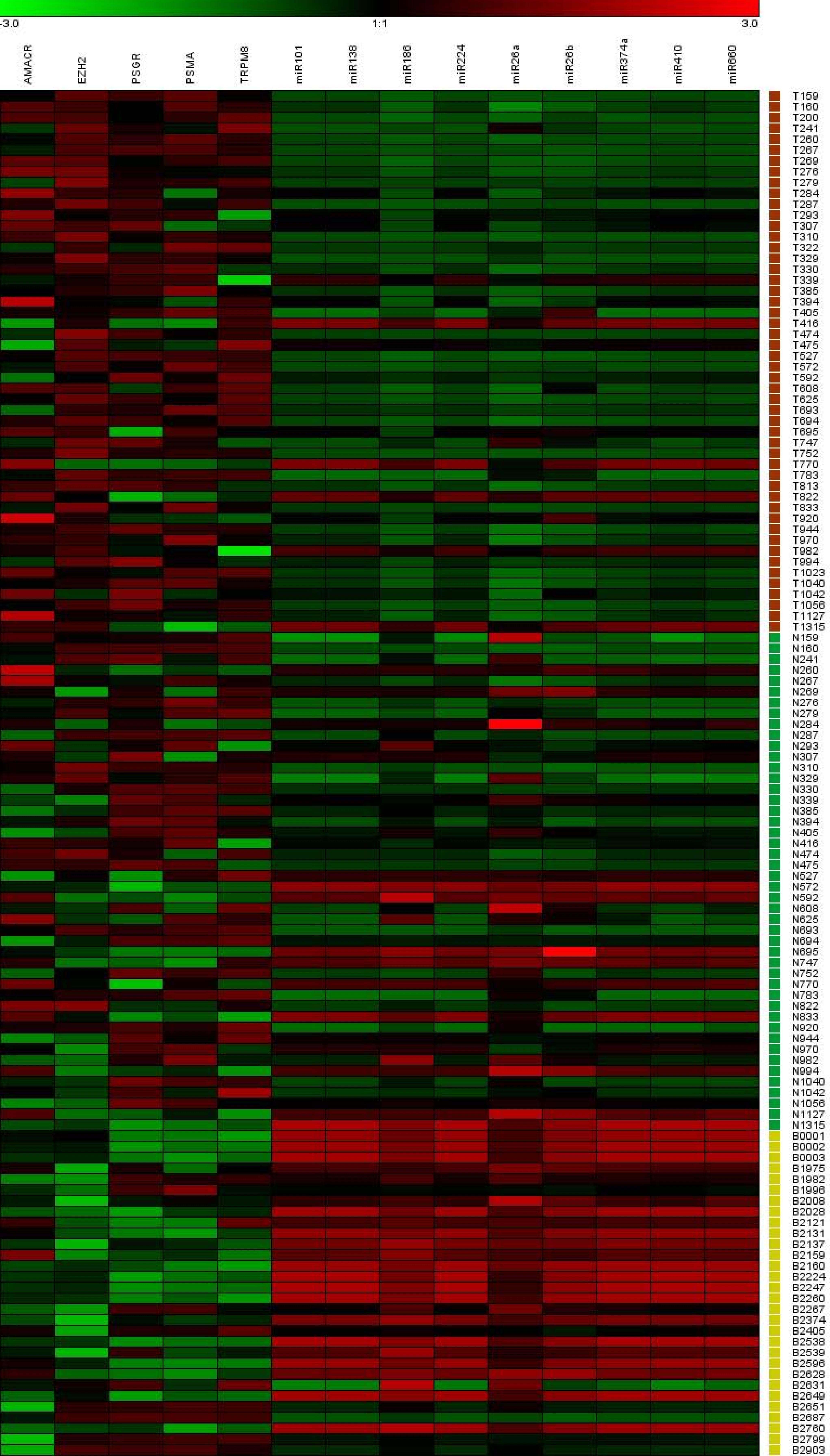
-3.0
1:1
3.0
AMACR
EZH2
PSGR
PSMA
TRPM8
miR101
miR138
miR186
miR224
miR26a
miR26b
miR374a
miR410
miR660
T159
T160
T200
T241
T260
T267
T269
T276
T279
T284
T287
T293
T307
T310
T322
T329
T330
T339
T385
T394
T405
T416
T474
T475
T527
T572
T592
T608
T625
T693
T694
T695
T747
T752
T770
T783
T813
T822
T833
T920
T944
T970
T982
T994
T1023
T1040
T1042
T1056
T1127
T1315
N159
N160
N241
N260
N267
N269
N276
N279
N284
N287
N293
N307
N310
N329
N330
N339
N385
N394
N405
N416
N474
N475
N527
N572
N592
N608
N625
N693
N694
N695
N747
N752
N770
N783
N822
N833
N920
N944
N970
N982
N994
N1040
N1042
N1056
N1127
N1315
B0001
B0002
B0003
B1975
B1982
B1996
B2008
B2028
B2121
B2131
B2137
B2159
B2160
B2224
B2247
B2260
B2267
B2374
B2405
B2538
B2539
B2596
B2628
B2631
B2649
B2651
B2687
B2760
B2799
B2903

Table S1: Web addresses of the bioinformatics resources used for miRNA prediction.

**Bioinformatic resource**	**Web address**
TargetScanHuman v5.1	http://www.targetscan.org/
TargetScanS	http://genes.mit.edu/tscan/targetscanS2005.html
PicTar<br><br>(based on conservation in mammals)	http://pictar.mdc-berlin.de/
MicroCosm Targets<br><br>(formerly miRBase Targets)	http://www.ebi.ac.uk/enright-srv/microcosm/htdocs/targets/v5/
microRNA.org	http://www.microrna.org/microrna/home.do
Human miRNA Targets<br><br>(optimized intersection: PicTar, TargetScanS)	http://www.diana.pcbi.upenn.edu/cgi-bin/TargetCombo.cgi
DIANA microT v3.0	http://diana.cslab.ece.ntua.gr/microT/
DIANA TarBase v5.0	http://diana.cslab.ece.ntua.gr/tarbase/

Table S2: Sequences of primers and probes used for qPCR.

Gene name (synonym)	Abbreviation (synonym)	Gene ID[a]	Primer / Probe	Sequence 5'-3'	Length of qPCR fragment	Ref.
alpha-methylacyl-CoA racemase	**AMACR**	23600	AMACR FOR<br>AMACR REV<br>AMACR FL<br>AMACR LC	gTATgCCCCgCTgAATCTC<br>TCCTTTgATCAgCAgCTCgTAg<br>ATgCTgTTCCTTCCACCATATTTgCATC-FL<br>LC640-ATgACCTgACCCTTgCCAgTgCgT-PH	312 bp	
enhancer of zeste homolog 2	**EZH2**	2146	EZH2 FOR<br>EZH2 REV<br>EZH2 FL<br>EZH2 LC	gCCAgACTgggAAgAAATCTg<br>TgTgTTggAAAATCCAAgTCA<br>AACCTCTTgAgCTgTCTCAgTCgCA-FL<br>LC640-TACTCTgATTTTACACgCTTCCgCC-PH	277 bp	[1]
prostate-specific G-protein coupled receptor (olfactory receptor, family 51, subfamily E, member 2)	**PSGR** (OR51E2)	81285	PSGR FOR<br>PSGR REV<br>PSGR FL<br>PSGR LC	AACTgCTgTATgggCTCTACTg<br>TgCCACTACATACATggAAAggAgg<br>TCCAgCTATgAgTTCCTgCAACTTCACAC-FL<br>LC640-TgCCACCTTTgTgCTTATTggTATCCCA-PH	179 bp	
prostate-specific membrane antigen (folate hydrolase 1)	**PSMA** (FOLH1)	2346	PSMA FOR<br>PSMA REV<br>PSMA FL<br>PSMA LC	CTTTCTCCTCggCTTCCTCTTC<br>gATTTTCATgTCCCgTTCCA<br>TTCCACTgggATTgAATTTgCTTTgC-FL<br>LC640-AgCTgAAAgTTTTgTTCTgTTCCTgCTAAATg-PH	485 bp	
TATA box binding protein	**TBP**	6908	TBP FOR<br>TBP REV<br>TBP FL<br>TBP LC	gAATATAATCCCAAgCggTTTg<br>ACTTCACATCACAgCTCCCC<br>TTTCCCAgAACTgAAAATCAgTgCC-FL<br>LC640-TggTTCgTggCTCTCTTATCCTCATg-PH	226 bp	[1]
transient receptor potential cation channel, subfamily M, member 8	**TRPM8** (Trp-p8)	79054	TRPM8 FOR<br>TRPM8 REV<br>TRPM8 FL<br>TRPM8 LC	ACgCTTgTgTACCggAATCT<br>CgAgTAATAggAgACACgTCg<br>TTTCCAgACAAACgTgAggAgggC-FL<br>LC640-CATTATAggAATTCTTggCgATCTgCA-PH	167 bp	[1-3]

[a] http://www.ncbi.nlm.nih.gov/gene

FL, fluorescence dye fluorescein; FOR, forward primer; LC640, fluorescence dye LC Red640; PH, phosphorylated 3'-end; REV, reverse primer

1. Schmidt U, Fuessel S, Koch R, Baretton GB, Lohse A, Tomasetti S, Unversucht S, Froehner M, Wirth MP, Meye A: **Quantitative multi-gene expression profiling of primary prostate cancer.** *Prostate* 2006, **66**(14):1521-1534.
2. Fuessel S, Sickert D, Meye A, Klenk U, Schmidt U, Schmitz M, Rost AK, Weigle B, Kiessling A, Wirth MP: **Multiple tumor marker analyses (PSA, hK2, PSCA, trp-p8) in primary prostate cancers using quantitative RT-PCR.** *Int J Oncol* 2003, **23**(1):221-228.
3. Kiessling A, Fussel S, Schmitz M, Stevanovic S, Meye A, Weigle B, Klenk U, Wirth MP, Rieber EP: **Identification of an HLA-A*0201-restricted T-cell epitope derived from the prostate cancer-associated protein trp-p8.** *Prostate* 2003, **56**(4):270-279.

Table S3: Conditions for qPCR measurements.

	**AMACR**	**EZH2**	**PSGR**	**PSMA**	**TBP**	**TRPM8**	**miRNAs**
**$MgCl_2$**	4 mM	4 mM	4 mM	4 mM	4 mM	4 mM	TaqMan Gene Expression Master Mix
**Primer** (for / rev)	0.5 µM each	0.5 µM each	0.5 µM / 0.25 µM	0.25 µM each	0.3 µM / 0.6 µM	0.5 µM each	Specific TaqMan MicroRNA Assays
**Probes** (FL / LC)	0.2 µM each	0.2 µM / 0.4 µM	0.2 µM each	0.2 µM / 0.4 µM	0.2 µM / 0.4 µM	0.2 µM each	
**Activation** (1 cycle)	95°C, 10 min	95°C, 10 min	95°C, 10 min	95°C, 10 min	95°C, 10 min	95°C, 10 min	95°C, 10 min
**Amplification** (45 cycles)							
**Denaturation step**	95°C, 10 s	95°C, 1 s	95°C, 1 s	95°C, 10 s	95°C, 10 s	95°C, 10 s	95°C, 15 s
**Annealing step**	59°C, 10 s	60°C, 15 s	59°C, 10 s	59°C, 15 s	61°C, 15 s	66°C, 15 s	60°C, 60 s
**Elongation step**	72°C, 10 s	72°C, 20 s	72°C, 10 s	72°C, 20 s	72°C, 10 s	72°C, 10 s	-

FL, probe with fluorescence dye fluorescein; for, forward primer; LC, probe with fluorescence dye LC Red640; rev, reverse primer

Table S4: Distributions of fold expressions of PCa-associated genes and miRNAs in prostate cancer. Depicted are the proportions of fold expression in Tu samples compared to either Tf or BPH samples. A fold expression of ≥2.0 was considered as up-regulation and of ≤-2.0 as down-regulation, whereas the remaining proportion was regarded as an unaltered expression.

**Gene / miRNA**	**Fold expressions: Tu *vs* Tf$_{[median]}$**			**Fold expressions: Tu *vs* BPH$_{[median]}$**		
	≤ -2.0-fold down n (%)	unaltered n (%)	≥ 2.0-fold up n (%)	≤ -2.0-fold down n (%)	unaltered n (%)	≥ 2.0-fold up n (%)
AMACR	5 (10%)	5 (10%)	40 (80%)	2 (4%)	4 (8%)	44 (88%)
EZH2	1 (2%)	29 (58%)	20 (40%)	0 (0%)	10 (20%)	40 (80%)
PSGR	9 (18%)	12 (24%)	29 (58%)	3 (6%)	2 (4%)	45 (90%)
PSMA	8 (16%)	14 (28%)	28 (56%)	2 (4%)	5 (10%)	43 (86%)
TRPM8	5 (10%)	15 (30%)	30 (60%)	2 (4%)	5 (10%)	43 (86%)
miR-101	22 (44%)	25 (50%)	3 (6%)	13 (26%)	32 (64%)	5 (10%)
miR-138	36 (72%)	12 (24%)	2 (4%)	36 (72%)	11 (22%)	3 (6%)
miR-186	43 (86%)	7 (14%)	0 (0%)	45 (90%)	5 (10%)	0 (0%)
miR-224	36 (72%)	14 (28%)	0 (0%)	34 (68%)	14 (28%)	2 (4%)
miR-26a	27 (54%)	23 (46%)	0 (0%)	22 (44%)	26 (52%)	2 (4%)
miR-26b	16 (32%)	29 (58%)	5 (10%)	12 (24%)	30 (60%)	8 (16%)
miR-374a	28 (56%)	21 (42%)	1 (2%)	23 (46%)	26 (52%)	1 (2%)
miR-410	35 (70%)	14 (28%)	1 (2%)	38 (76%)	11 (22%)	1 (2%)
miR-660	19 (38%)	30 (60%)	1 (2%)	20 (40%)	29 (58%)	1 (2%)

Table S5: Expression of selected genes in prostate cancer dependent on clinicopathological parameters. Depicted are the median relative transcript levels of the evaluated genes (normalized to TBP) in Tu tissue samples. A Mann–Whitney *U* test with a two-sided 95% confidence interval was used for two group comparisons. Three-group comparisons were carried out by the Kruskal–Wallis test followed by a Mann–Whitney *U* test with a two-sided 95% confidence interval for *post hoc* analyses. Significant differences are highlighted in bold.

**Parameter**	**AMACR**	**EZH2**	**PSGR**	**PSMA**	**TRPM8**
	**P values and median relative transcript levels**				
**Age**	p = 0.635	p = 0.778	p = 0.114	p = 0.869	p = 0.165
≤ median (n=25)	2228.90	0.99	67.79	29.56	52.31
> median (n=25)	1771.34	0.90	25.02	15.76	31.18
**PSA**	p = 0.705	p = 0.705	p = 0.079	p = 0.869	p = 0.308
≤ median (n=25)	2341.57	0.99	67.79	16.91	31.85
> median (n=25)	1935.56	0.90	38.70	29.49	52.31
**Tumor stage**	p = 0.915	p = 0.661	p = 0.247	p = 0.676	**p = 0.030**
pT1+2 (n=23)	2341.57	0.99	66.95	15.89	52.44
pT3+4 (n=27)	1935.56	0.90	38.70	28.03	25.14
**Gleason score**	p = 0.706	p = 0.354	p = 0.128	p = 0.906	p = 0.421
< 7 (n=16)	2369.88	0.86	65.35	25.88	38.16
7 (n=19)	1303.14	0.99	60.30	28.01	41.13
> 7 (n=15)	1771.34	0.90	15.69	28.03	16.49
**Initial metastases**	p = 0.225	p = 0.410	p = 0.160	p = 0.297	p = 0.182
N0 M0 (n=40)	2339.05	0.98	50.89	25.88	38.55
N+/M+ (n=10)	969.76	0.84	43.31	28.02	27.56

Table S6: Expression of selected miRNAs in prostate cancer dependent on clinicopathological parameters. Depicted are the median relative transcript levels of the evaluated miRNAs (normalized to RNU48) in Tu tissue samples. A Mann–Whitney *U* test with a two-sided 95% confidence interval was used for two group comparisons. Three-group comparisons were carried out by the Kruskal–Wallis test followed by a Mann–Whitney *U* test with a two-sided 95% confidence interval for *post hoc* analyses. Significant differences are highlighted in bold.

Parameter	miR-101	miR-138	miR-186	miR-224	miR-26a	miR-26b	miR-374a	miR-410	miR-660
	P values and median relative transcript levels (x10-3)								
**Age**	p = 0.388	p = 0.643	p = 0.877	p = 0.915	p = 0.299	p = 0.091	p = 0.509	p = 0.090	p = 0.432
≤ median (n=25)	1.57	0.24	36.60	1.12	194.32	53.32	17.91	0.12	13.60
> median (n=25)	1.47	0.15	40.49	1.27	149.10	35.59	17.24	0.00	11.87
**PSA**	p = 0.662	p = 0.657	p = 0.907	p = 0.432	p = 0.607	p = 0.786	p = 0.218	p = 0.572	p = 0.720
≤ median (n=25)	1.56	0.24	38.43	1.38	194.32	51.32	19.88	0.00	14.84
> median (n=25)	1.47	0.14	40.49	0.99	165.54	52.95	16.24	0.09	13.32
**Tumor stage**	p = 0.778	p = 0.262	**p = 0.028**	p = 0.838	p = 0.459	p = 0.969	p = 0.414	p = 0.635	p = 0.711
pT1+2 (n=23)	1.47	0.43	47.70	1.27	149.62	51.50	20.02	0.00	11.42
pT3+4 (n=27)	1.59	0.13	33.89	1.12	194.32	52.04	16.82	0.09	13.98
**Gleason score**	p = 0.633	p = 0.866	p = 0.742	p = 0.721	p = 0.752	p = 0.495	p = 0.913	p = 0.914	p = 0.786
< 7 (n=16)	1.48	0.39	41.37	1.43	194.66	50.70	18.36	0.11	14.92
7 (n=19)	1.56	0.15	38.03	1.08	175.64	52.95	17.91	0.09	11.95
> 7 (n=15)	1.44	0.15	40.49	0.93	149.10	51.50	17.36	0.00	13.98
**Initial metastases**	p = 0.370	p = 0.480	**p = 0.005**	p = 0.369	p = 0.528	p = 0.865	p = 0.913	p = 0.625	p = 0.445
N0 M0 (n=40)	1.47	0.34	44.86	1.30	177.19	51.77	17.63	0.05	11.91
N+/M+ (n=10)	1.66	0.07	31.18	0.89	194.32	52.13	17.80	0.09	14.92
